# Non-signaling but all important: how the linker, hinge, and transmembrane domains in the CAR hold it all together

**DOI:** 10.3389/fimmu.2025.1664403

**Published:** 2025-10-27

**Authors:** Grace Bernard, Laura Evgin

**Affiliations:** ^1^ Interdisciplinary Oncology Program, University of British Columbia, Vancouver, BC, Canada; ^2^ Basic and Translational Research Department, BC Cancer Research Institute, Vancouver, BC, Canada; ^3^ Department of Medical Genetics, University of British Columbia, Vancouver, BC, Canada

**Keywords:** chimeric antigen receptor (CAR), T cell, transmembrane domain (TMD), hinge domain, linker region

## Abstract

The chimeric antigen receptor (CAR) is a synthetic and modular molecule composed of both signaling and non-signaling domains that allows a T cell to recognize cell surface antigens and trigger cytolytic functionality. It is appreciated that the non-signaling structural components, including the linker, hinge, and transmembrane domains, can dramatically alter how the CAR molecule interacts with itself and other endogenous molecules in the immune synapse. Herein, we describe the current understanding of how the structural domains can alter CAR T cell therapeutic efficacy and highlight how knowledge of the target antigen characteristics can inform CAR design choices.

## 1 Introduction

Chimeric antigen receptor (CAR) modified T cells have provided a paradigm shift in the management of hematological cancers, and there are seven Food and Drug Administration (FDA) approved therapies for relapsed/refractory acute lymphoblastic leukemia (ALL) (1–3), aggressive B cell lymphoma (4–6), mantle cell lymphoma (7), indolent B cell lymphoma (8, 9), and multiple myeloma (10–12). CAR T cells targeting CD19 and BMCA have induced prolonged remissions in patients with advanced malignancies with minimal long-term toxicities, and this success has been facilitated by the lineage-restricted and uniform expression of these antigens (13). CD19 and BCMA-specific CAR T cells are also being repurposed for the treatment of autoimmune diseases, where autoreactive cells of the B cell lineage (B cells themselves, plasmablasts, and plasma cells) are central mediators of disease pathology (14, 15).

## 2 Overview of the architecture of a CAR

**Figure 1:**
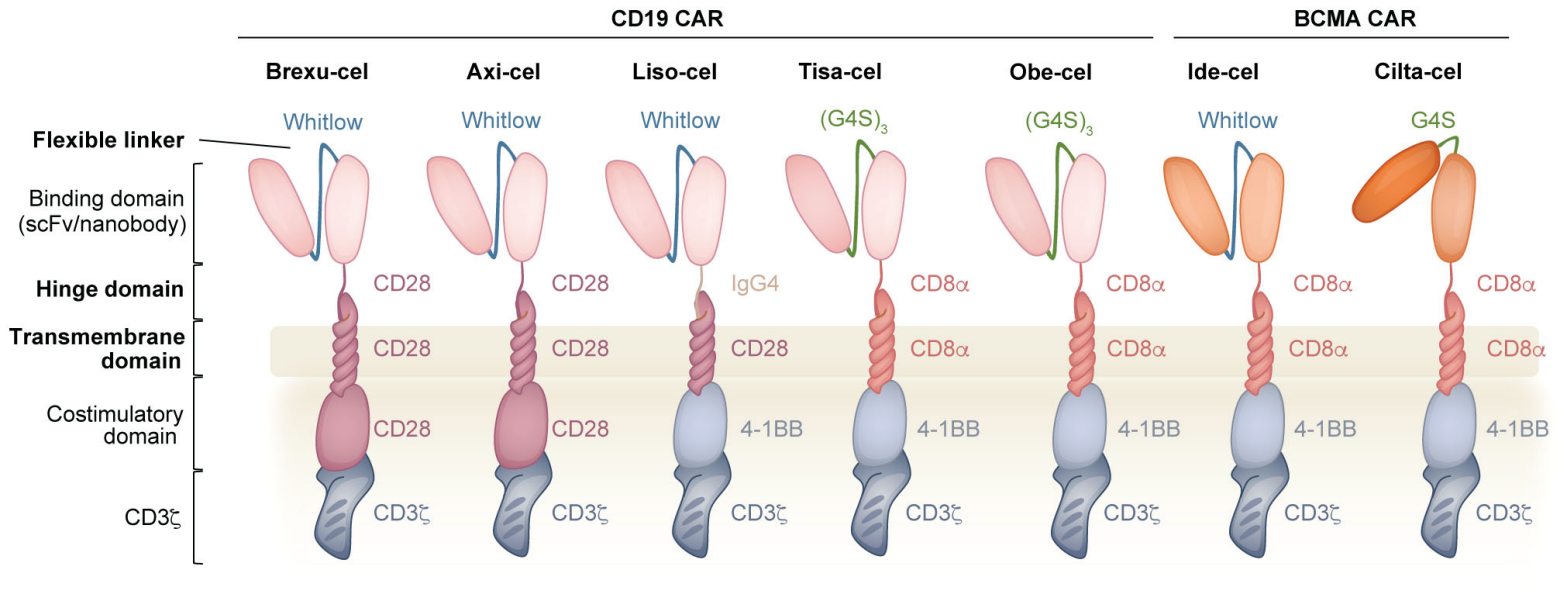
Overview of the CAR and the components that are used in clinically approved CD19 and BCMA CAR T cell products. The binding domain of the CAR comprises an scFv (or nanobody) where the V_H_ and V_L_ (or two V_H_ domains) are connected via a flexible linker (Whitlow or (G4S)_n_). The hinge domain (derived CD28 or CD8α or IgG4) connects the binding domain to the transmembrane domain (derived CD28 or CD8α). In the intracellular region, the costimulatory domain (derived from CD28 or 4-1BB) is connected to CD3ζ. Bolded domains are described in the text.

The synthetic CAR molecule brings together the recognition capabilities of an antibody with the signaling properties of the T cell receptor (TCR) complex to redirect T cell function in a TCR-major histocompatibility complex (MHC) independent manner (**Figure 1**). The ectodomain contains a binding region responsible for recognizing the target cell antigen, which is frequently derived from a single-chain variable fragment (scFv) or a camelid nanobody (16, 17). However, specificity can also be conferred by a variety of natural ligands (18, 19), receptors (20), short peptides (21), or even fully synthetic binders, such as the D-domain (22). This recognition domain is fused to the hinge and transmembrane domains which provide flexibility and embed the protein in the membrane, respectively (23). The TCR α and β subunits do not have signaling properties but rather associate with CD3 chains. Thus, to mimic the TCR signal, the cytosolic portion of CD3ζ is fused at the distal end of the CAR molecule, where three (or fewer) (24, 25) ITAMs are involved in an LCK-mediated phosphorylation cascade involving LAT, SLP-76, and PLCγ; ultimately resulting in CAR T cell activation (26). Since effective T cell responses require both a primary signal from the TCR, as well as a secondary signal from a costimulatory molecule, the endo domain also includes the cytosolic region of one or more costimulatory molecules, membrane-proximal to the CD3ζ domain. While clinically approved CAR T cells incorporate CD28 or 4-1BB, many alternatives have been explored, including ICOS (27), OX40 (28), CD2 (29), CD27 (30), and IL-2RB in combination with a STAT3 binding motif (31), and shuffling these costimulatory domains alters the kinetic, differentiation, persistence, and cytolytic properties of CAR T cells (32, 33).

## 3 Non-signaling components in the CAR

Beyond the hematological targets CD19 and BCMA, the modularity of the CAR system allows for applications in solid tumors, infectious and autoimmune diseases, aging, and fibrosis, etc., by a simple substitution of domains (34–38). Whether designing a novel CAR, or optimizing the sensitivity or function of an existing CAR, many possible permutations exist given the library of previously described domains. While the recognition and signaling domains have previously been extensively reviewed (39–41), herein, we describe the origin, nature and functional characteristics imparted by the non-signaling components of the CAR, including the linker, hinge and transmembrane domains. Moreover, we explore how some structure-activity relationships may be generalized, whereas others are construct, antigen, or epitope-specific. Finally, we propose open questions to make informed receptor design choices and guide future work. While these domains are scaffold components of the CAR, they profoundly shape CAR T cell functional properties and thus therapeutic outcomes. Finally, our review focuses on the design of CARs for expression in T cells, but we acknowledge that these synthetic molecules can redirect other cell types, including NK cells and macrophages, and the optimal structural elements for these cellular substrates differ. For example, in NK cells, a CAR containing the NKG2D transmembrane domain displayed superior functionality to the CD28 transmembrane domain (42).

### 3.1 Linker region

In CARs that incorporate an scFv as the mode of recognition, the linker is a synthetic peptide that covalently joins the variable heavy (V_H_) and light (V_L_) chains. One commonly used linker is composed of a pentapeptide series of four glycine residues followed by serine and is repeated three or four times as a 15-mer (G4S)_3_ or 20-mer (G4S)_4_ (43). The small size of these residues provides flexibility, and the polar nature of serine increases linker solubility, limiting interference with the variable region folding and target binding (44). Alternatively, the 218 linker, commonly known as the Whitlow linker (GSTSGSGKPGSGEGSTKG), is also frequently used and was designed to have reduced susceptibility to aggregation and proteolysis compared to the earlier 202 linker (45–47). Both linkers are used in clinically approved products: Whitlow (Brexu-cel, Liso-cel, Axi-cel and Ide-cel); G4S (Tisa-cel, Obe-cel and Cilta-cel) (**Figure 1**) (48, 49). Antibodies with specificity to these linkers facilitate both the detection of surface expression (48, 50, 51) and the purification (52) of CAR-expressing cells.

#### 3.1.1 Identity of the linker

As they are similar in length, it is not clear whether the choice of the G4S or Whitlow linker imparts significant differences on CAR T cell functional outcome, nor has this been extensively reported on. Kouro et al. compared the use of the Whitlow and (G4S)_4_ linkers with an scFv with a propensity for tonic signaling in the context of both 4-1BB and CD28 co-stimulation. No differences were observed in surface aggregation, NFAT and NFκB signaling intensity, or *in vivo* tumor control in a xenograft model. However, the Whitlow-linked CAR did produce higher levels of IL4, TNFα, GM-CSF and IL10 in a costimulatory domain-specific manner (53).

#### 3.1.2 Length of the linker

The properties conferred on an scFv by the length of the linker have been investigated in detail (54). An scFv with a linker longer than 12 amino acids exists as a monomer whereas a linker of 3 to 12 residues promotes association with a second scFv molecule to produce a “diabody” (55–58). In turn, short linker-based multivalent scFvs have greater binding valency to target molecules on the cell surface and reduced off-rates compared to monovalent scFvs (59, 60).

In the context of a CAR, Singh et al. examined the efficacy of two CD22-targeting CARs that differed only in the length of their linker: a CD22 long linker consisting of four G4S repeats (CD22-L) or a short linker composed of a single G4S (CD22-S) (61). This investigation was prompted by discrepant clinical outcomes using CAR T cells incorporating the same V_H_ and V_L_ chains, albeit connected via linkers of different lengths (62). The CD22-S CAR molecule was found to aggregate and drive low-level antigen-independent tonic signaling, as measured by activation of the phosphotidyl-inositol-3 kinase (PI3K) and mitogen-activated protein kinase (MAPK) signaling pathways. Compared to the CD22-L CAR T cells, the CD22-S CAR T cells also remained in contact longer with target cells, consistent with the slower off-rate of scFv multimers, producing more effector cytokines and demonstrating superior killing both *in vitro* and *in vivo*. Although not experimentally tested in this study, the authors suggest that shortening the linker to enhance affinity could be a useful design strategy for targeting antigens expressed at low density. These advantages were observed with the 4-1BB but not CD28 costimulatory domain-based CAR, in line with previous reports that the CD28 costimulatory domain exacerbates tonic signaling-associated dysfunction (63, 64). The enhanced tonic signaling and antigen-dependent activation conferred by the short hinge were also observed with CD33 but not CD19-based CARs. Therefore, while shortening the linker may promote clustering for some V_H_ and V_L_ domain pairings, this does not represent a universal strategy to enhance CAR T cell activity, as susceptibility to clustering and tonic signaling is also determined by biochemical properties of the framework region (65).

### 3.2 Hinge domain

The hinge domain, also known as the spacer region, connects the binding domain to the transmembrane domain, and provides the flexibility for the CAR to interact with a specific antigen of interest. The most commonly used hinges are derived from CD8α and CD28, however, IgG molecules have also been used preclinically and clinically (**Figure 1**) (13). Nerve growth factor receptor (NGFR) and CD34, two molecules normally absent from mature T cells, have also been used as alternative hinge domains to facilitate the detection of the CAR and the immunomagnetic sorting of CAR T cells (66, 67). The properties of the hinge, including its identity and length, shape how the CAR responds to antigen density and epitope position, ultimately affecting sensitivity and signaling strength.

#### 3.2.1 Identity of the hinge

The hinge domain retains features of the native molecule, which affect the functional properties of the CAR. For example, CD28 typically exists as a homodimer due to an interdomain disulfide bond (68, 69), and the critical cysteine at position 123 is incorporated into the CAR hinge domain. A series of CD19 FMC63-based CARs incorporating various hinge and transmembrane domains identified that the cysteine in the CD28 hinge domain can stabilize a heterodimer of the CAR and endogenous CD28 (further described in the transmembrane section below) (70). Similarly, CD8α is expressed on T cells as a mixture of CD8αα homo- and CD8αβ heterodimers, with dimerization mediated by Ig domain interactions in the ectodomain as well as disulfide bonds formed by cysteine residues in the hinge and transmembrane regions (71, 72). The CD8α-derived hinge region incorporated into the CAR is known to be intrinsically disordered and dynamically transitions between conformation states involving proline cis–trans isomerization. A CD8α hinge-based CAR targeting CD22 was found to outperform a CD28 hinge-based CAR *in vitro* against low antigen density leukemia, and it was suggested that the flexibility of the CD8α hinge, driven by cis-trans isomerization, in combination with disulfide bridging between dimeric molecules, enhanced the signal transmission and sensitivity of the CAR (72). In contrast to the disorder of the CD8α hinge, the structural rigidity of the IgG4 hinge, in combination with two embedded cysteine residues, was found to promote homodimerization and the interaction of camelid V_H_ domains in a GPC1 CAR to amplify T cell signaling (73). Finally, IgG4 or IgG1-derived hinges containing the CH2-CH3 domain retain the ability to interact with the Fc receptor, and while these CAR T cells perform well *in vitro*, *in vivo*, they lacked persistence and therapeutic activity, likely due to interaction with Fc-receptor-bearing myeloid cells. However, T cells equipped with a CAR bearing either a deletion of the CH2 domain, which interacts with the Fc receptor, or a specific mutation of the involved residues (L235E, N297Q) within the CH2 region, exhibited improved persistence and elicited more potent anti-tumor efficacy in mice (74–76).

#### 3.2.2 Length of the hinge

**Figure 2:**
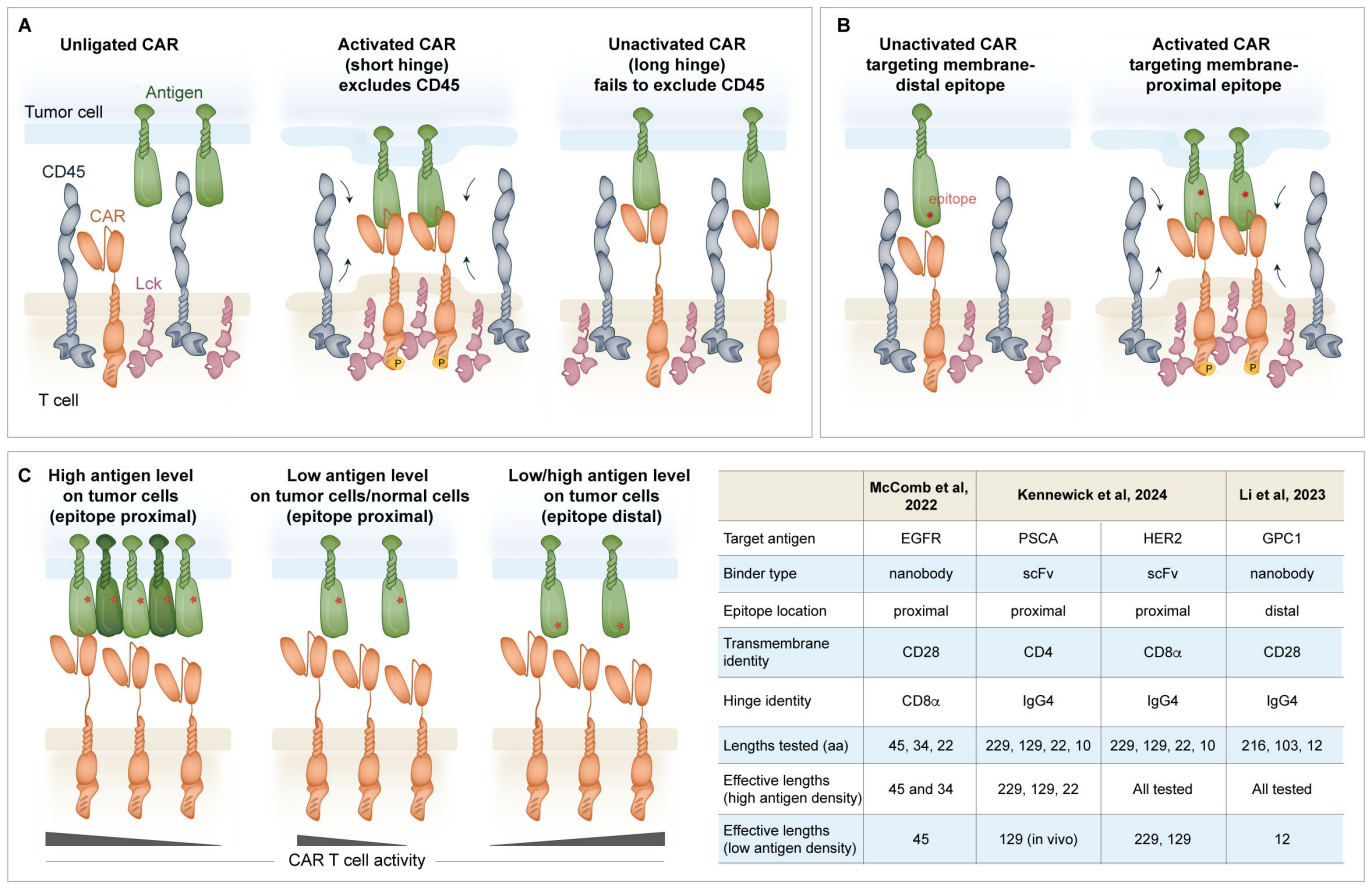
Regulation of the immune synapse formation between the CAR and the antigen of interest. **(A)** Effective activation of a CAR T cell is described by the “size exclusion model” where antigen binding by the CAR narrows the intermembrane space and excludes the CD45 phosphatase from the CAR zone, favoring Lck phosphorylation of the ITAMs in CD3ζ. If the hinge is too long, CD45 is ineffectively excluded (80). **(B)** The location of the epitope on the antigen of interest is important. scFvs targeting proximal epitopes may be better suited than those targeting distal epitopes to trigger the immune synapse. **(C)** The hinge can be shortened to increase the selectivity of the CAR for cells with high antigen density. This is useful for CARs that recognize shared antigens (i.e. HER2 and others) that are expressed at a lower level on normal cells. The hinge may also be shortened to accommodate binders that target membrane distal epitopes to promote effective formation of the immune synapse.

The length of the hinge contributes to the overall functionality of a CAR in an antigen- and epitope-specific manner. The “kinetic segregation” model was originally proposed to describe peptide-MHC-TCR activation, where exclusion of the CD45 tyrosine phosphatase from the TCR signalosome shifts the equilibrium in favor of Lck-mediated TCR complex phosphorylation (77–79). In an analogous manner, Xiao et al. proposed the “size exclusion” model for CAR triggering where antigen binding narrows the intermembrane space and segregates the bulky CD45 phosphatase from the CAR zone, thus facilitating phosphorylation (**Figure 2A**) (80). Although described with different terminology, both models invoke the same principle where binding creates spatial constraints that exclude CD45 and enable phosphorylation. Using a CD19-targeting CAR with various hinge lengths, the size of the extracellular domain was determined to be inversely proportional to CD45 exclusion from the immune synapse, CD3ζ and ERK phosphorylation, cytokine production, and *in vitro* killing. This size-dependent activation also held true *in vivo*, where the shorter hinge CAR constructs provided superior tumor control in xenograft mouse models. To contextualize these findings, the CD8α hinge was used as the base construct, and additional Ig domains were added to increase the length by 4–16 nm. Consistent with this model where a shorter intermembrane distance elicits stronger exclusion of CD45, using CD22- and CEA-specific CARs, it was also shown that membrane-proximal epitopes stimulate CAR T cell activation better than distal epitopes (80). This new framework also helps explain previous reports showing that CARs with scFvs targeting membrane-proximal epitopes exhibit superior functional properties compared to those targeting distal epitopes (**Figure 2B**) (81, 82).

Given these implications on activation, together with knowledge of the target epitope location and antigen density, the length of the hinge can be manipulated to fine-tune CAR sensitivity (**Figure 2C**). The selection of shorter hinges can promote antigen-driven activation of CARs targeting membrane distal epitopes (73, 83). Conversely, shortening the hinge length is a useful method to attenuate the activation of a CAR targeting a membrane-proximal epitope of an overexpressed but not cancer-specific antigen, such as epidermal growth factor receptor (EGFR), human epidermal growth factor receptor 2 (HER2), or prostate stem cell antigen (PSCA) (84, 85). While tumor cells with high antigen density elicited the activation of HER2 CAR T cells with a conventional 45 amino acid CD8α hinge as well as the 34 amino acid truncated hinge, tumor cells with low antigen density selectively activated the longer hinge bearing CAR (84). Similarly, PSCA-targeted CAR T cells incorporating a 229 or 129-amino acid IgG4-derived hinge activated against high and low antigen targets, whereas a CAR containing a 22-amino acid long IgG4 hinge only displayed activity against high antigen targets (85). This mechanism of selectivity is likely independent of the size exclusion model, as CARs with the longest hinge are still capable of activation (i.e., all CARs can exclude CD45). Instead, other structural or steric factors, such as restricted flexibility or suboptimal scFv orientation when the hinge is too short, may influence activation. Although this approach tunes the safety and selectivity profile of the CAR, it may also have the unwanted effect of selecting for tumor variants with even lower antigen density that can escape CAR T cell activation.

Taken together, these results underscore that there is not a one-size-fits-all approach to hinge engineering and that optimization is required for each CAR. Nonetheless, understanding the properties of the scFv (i.e. affinity, oligomerization propensity), the position of the target epitope on the antigen and its proximity to the membrane, as well as overall antigen density may help to predict a ‘goldilocks’ hinge identity and length.

### 3.3 Transmembrane domain

The transmembrane domain links the extracellular region to the intracellular signaling domains and anchors the CAR to the surface of the cell. The transmembrane domain from CD8α and CD28 are the most popular choice preclinically and clinically (**Figure 1**), however, CD3ζ and CD4 transmembrane domains have also been described (86, 87). Although in some cases the hinge and transmembrane domains are studied separately, they are often used *en bloc*, thereby making it difficult to distinguish the functional contributions of each domain.

#### 3.3.1 Surface expression

Fujiwara et al. investigated the roles of the hinge and transmembrane domains in regulating the surface expression of the CAR using domains from CD4, CD8α, and CD28 (87). T cells transduced with a VEGFR2-specific CAR with a hinge and transmembrane domain from CD8α or CD28 showed higher surface expression than CARs with a hinge and transmembrane domain derived from CD3ζ or CD4, suggesting these domains play an important role in the stability of surface presentation (87). Similar findings were also observed with an NKp30 CAR where CD8α or CD28 transmembrane domains provided superior surface expression compared to a CD3ζ transmembrane domain (88).

#### 3.3.2 Association with endogenous molecules

Not only are transmembrane domains actively involved in regulating surface expression, but they are also capable of mediating interactions with endogenous proteins. A first-generation carcinoembryonic antigen (CEA)-specific CAR containing a CD3ζ-derived transmembrane domain was found to dimerize and form complexes with endogenous TCRs (86). As noted in the section on the hinge domain, CARs with a CD28 transmembrane domain, but not those with a CD8α transmembrane domain, were shown to heterodimerize with endogenous CD28 (70). This interaction was demonstrated by co-immunoprecipitation studies, as well as stimulation with anti-CD28, which elicited CAR-dependent proliferation. This phenomenon was attributed to four polar amino acids found in the CD28 transmembrane domain, and disruption of these amino acids abrogated the interaction (70).

#### 3.3.3 Regulation of functional sensitivity

To address the issue of CAR T cell evasion by antigen-low tumor variants, Majzner et al. explored how the CD8α or CD28 hinge and transmembrane domains affect the cytolytic properties of an FMC63-based CD19 CAR (89). Although functionally equivalent in a high antigen density setting, CARs containing the CD8α hinge/transmembrane displayed reduced killing and cytokine production in an antigen low setting compared to the CD28 hinge/transmembrane CAR. The T cells with the CD28 hinge/transmembrane CAR killed their targets more quickly post-engagement, and this was attributed to their ability to form microclusters, followed by supramolecular activation clusters (cSMACs), and recruit ZAP70. In this way, the hinge/transmembrane domain can tune the threshold for antigen recognition to enhance efficacy against antigen low targets (89).

#### 3.3.4 Toxicity implications of hinge/transmembrane choices

Both anti-CD28 stimulation, as well as cell-based expression of the natural ligands of CD28, CD80 and CD86, have been shown to activate T cells expressing high levels of CD28 transmembrane-based CARs, raising the concern of off-target activation (70). Although co-stimulation blockade with CTLA4-Ig was shown to block this effect *in vitro*, and may also provide a therapeutic solution, further investigation is warranted to understand the implications of this phenomenon. CAR T cell–mediated tumor killing may activate antigen-presenting cells (APCs), such as macrophages, to upregulate CD80/86, creating a positive feedback loop that drives antigen-independent proliferation and function of CAR T cells containing the CD28 hinge and transmembrane domains, thereby contributing to cytokine release syndrome (CRS). Similar mechanisms may arise in other clinical settings, such as infectious complications, where innate immune activation induces CD80/86 expression on APCs, potentially stimulating these CAR T cells. Because this effect was most pronounced when CAR surface expression was very high, engineering strategies such as targeted CAR insertion into the TRAC locus may help limit expression levels and reduce this risk (90).

Alabanza et al. showed that for two CD19 targeting CARs (FMC63 and a humanized scFv), those incorporating CD8α hinge and transmembrane domains produced lower levels of cytokines and exhibited less activation-induced cell death *in vitro* versus CARs incorporating the CD28 hinge and transmembrane domains (91). Because cytokine-mediated toxicity is a key factor in managing patient care after CAR T cell therapy, the authors propose that selecting CARs with reduced cytokine release profiles may represent a favorable design strategy. Indeed, clinical testing also demonstrated that the CD8α hinge and transmembrane domain containing CAR (Hu19-CD828ζ) exhibited fewer neurologic toxicities and serum cytokine levels than historical use of the CD28 hinge and transmembrane containing CAR (FMC63–28ζ) (92).

## 4 Open questions

- What is the degree of generalizability of the above-mentioned findings? How does the integration of the sum of the components attenuate or exacerbate trends? For example, shortening the hinge has been shown to increase selectivity for highly expressed antigen targets with proximal binding epitopes (**Figure 2C**). However, precisely how long or short the hinge should be differs between studies, suggesting that the other features of the CAR and the epitope also determine the threshold.
  ○ McComb et al. consider the full-length 45 amino acid CD8α hinge to be long, whereas Li et al. consider the 226 amino acid IgG4 hinge to be long (73, 84). In the McComb et al. study, functionality fell off in the low antigen setting between 34 and 22 amino acids, and in the Li et al. study, functionality was limited at 22 amino acids. In the low antigen setting where the CAR targets a proximal antigen, a variety of “longer” hinges may be tolerated, but an activation cut-off may converge across constructs in the range of 30 amino acids.
- When setting out to develop a new CAR, it is not possible to try all permutations of linker, hinge and transmembrane domains, so what combination of pieces might mitigate risk to develop a highly sensitive CAR? Should binder campaigns prioritize validation of scFvs targeting membrane-proximal epitopes? Should membrane-distal parts of the antigen not be part of the bait molecule used to identify binders? Should the CD28 hinge/transmembrane be tested first, as it provides the greatest sensitivity?
- Is shortening the linker a general strategy to increase the functional avidity of the CAR, particularly for antigenic targets that are not abundantly expressed? Singh et al. might suggest that only some scFv/epitope combinations are amenable to this approach. Depending on the antigen binding site, the orientation may or may not be amenable to a CAR dimer binding multiple targets. Although a 4-1BB costimulatory domain may be required to avoid dysfunction, are other engineering approaches also essential to elicit synthetic T cell states to accommodate low-level tonic signaling (93)?
- How does the hinge length and flexibility affect the ability of a CAR to bind in *cis* and exert either protective effects where the target is expressed on the T cell (94), or epitope masking on tumor cells (95)? This flexibility and masking property is likely to be similarly determined by the size of the antigen and the epitope location.
- Will these properties established in *ex vivo* engineered T cells be translatable, or do accommodations need to be made for *in vivo* engineered CARs where the molecule is transiently expressed by RNA? A CD28 hinge/transmembrane configuration was preferred over the CD8α-derived domain in a study testing mRNA/lipid nanoparticle (LNP) *in vivo* engineering, although this comparison was not investigated in depth (96).
- CARs are also being expressed in other cell types, including macrophages and NK cells. How the hinge and transmembrane domain can be tailored for these settings is only beginning to be explored.

## 5 Discussion

Seemingly minor structural changes to a CAR can impart strong functional characteristics. To design an efficient receptor, a deep understanding of the individual CAR modular elements, the characteristics of the antigen of interest, and the intersection of these properties is required. Decisions about which structural components to select can have profound consequences regarding the susceptibility to tonic signaling, interaction with or exclusion of endogenous molecules from the signalosome, and can tune the signal strength and threshold of required antigen density for activity. However, as the biochemical design rules are being uncovered, the degree to which they are broadly applicable or unique to an experimental context needs to be recognized.

Not all antigens are created equal. They may have a wide or narrow window of tumor selectivity. They may be densely or sparsely expressed and have variable propensities to reduce expression in response to CAR selective pressure. The specific location of the binding epitope on the antigen of interest is also a key variable. All these factors can be experimentally considered in the construction of a CAR. For example, low antigen density has been addressed by inducing tonic signaling with short linkers to prime the CAR T cells to respond when needed (61) and by the incorporation of the CD28 hinge and transmembrane domains, which have superior sensitivity to coordinate an organized immune synapse. Finally, scFvs targeting membrane distal epitopes can be accommodated by shortening the length of the hinge region (73) to tighten the intermembrane space and exclude the CD45 phosphatase (80).

Taken together, the synthetic CAR molecule delivered as a cell therapy has revolutionized the way we think about disease treatment and immunotherapy. CARs are celebrated for their modular nature, and the choice of each component represents an opportunity to maximize function and tailor the therapy to the unique context.
